# Variability in SCC*mec*_N1 _spreading among injection drug users in Zurich, Switzerland

**DOI:** 10.1186/1471-2180-7-62

**Published:** 2007-07-02

**Authors:** Miriam Ender, Brigitte Berger-Bächi, Nadine McCallum

**Affiliations:** 1 Institute of Medical Microbiology, Gloriastr. 32, CH8006 Zurich, University of Zurich, Switzerland

## Abstract

**Background:**

An extremely low level methicillin resistant *Staphylococcus aureus *(MRSA) belonging to ST45, circulates among intravenous drug users in the Zurich area. This clone can be misinterpreted as an MSSA by phenotypic oxacillin resistance tests, although it carries a staphylococcal cassette chromosome *mec *(SCC*mec*) element encoding a functional *mecA *gene and it produces PBP2a.

**Results:**

This clone carried a new 45.7-kb element, termed SCC*mec*_N1_, containing a class B *mec *complex (*mecA-*Δ*mecR1::IS1272*), a truncated Tn*4003 *harbouring the *dfrA *gene, and a *fusB1 *gene, conferring methicillin, trimethoprim and low level fusidic acid resistance, respectively. In addition to the two insertion site sequences (ISS) framing the SCC*mec*, a third ISS (ISS*) was identified within the element. SCC*mec*_N1 _also harboured two distinct *ccrAB *complexes belonging to the class 4 subtype, both of which were shown to be active and to be able to excise the SCC*mec*_N1 _or parts thereof. Slight variations in the SmaI-PFGE pattern of the clinical MRSA isolates belonging to this clone were traced back to differences in the sizes of the SCC*mec *J2 regions and/or to a 6.4-kb deletion extending from ISS* to the right end ISS. This latter deletion led to a variant right SCC*mec*-chromosomal junction site. MRSA clones carrying the shorter SCC*mec *with the 6.4-kb deletion were usually ciprofloxacin resistant, while strains with the complete SCC*mec*_N1 _were co-trimoxazole resistant or had no additional resistances. This suggested that the genetic backbone of the host *S. aureus*, although identical by PFGE pattern, had at some stage diverged with one branch acquiring a sulfonomide resistance mutation and the other ciprofloxacin resistance.

**Conclusion:**

This description of the structure and variations of SCC*mec*_N1 _will allow for quicker and easier molecular detection of this clone and monitoring of its spread.

## Background

Injection drug user (IDU) populations throughout certain areas of Europe and North America have become major risk groups associated with the epidemic spread of methicillin-resistant *Staphylococcus aureus *(MRSA) [1-5]. The transmission of MRSA clones through both community- and healthcare-associated routes is responsible for the high incidence of soft tissue infections and increases in severe infections such as endocarditis and bacteremia in IDUs [6-8]. Such a clonal dispersal led to MRSA becoming endemic in the Zurich IDU population, where in 2003 24% of all MRSA isolates collected at the University hospital of Zurich belonged to a single, so called "drug clone" [9]. Dissemination of this clone to IDU populations in other, geographically distinct regions of Switzerland has also been recently reported, indicating that it has a capacity for spreading and colonizing new populations [10].

Clinical detection of MRSA can be complicated due to vast strain-to-strain differences in the expression of methicillin resistance. Difficulties arise from strains expressing low-level but heterogenous resistance, that upon exposure to β-lactams segregate highly resistant subpopulations resulting in therapy failure [11]. Misdiagnosis of such strains with very low, phenotypically susceptible, minimum inhibitory concentrations (MICs) is a major problem necessitating the use of molecular detection methods.

Epidemiological classification of MRSA strains is important for monitoring their prevalence and spread, and relies on molecular typing of both their core genomic background and the type of staphylococcal cassette chromosome *mec *(SCC*mec*) they harbour. SCC*mec *is the chromosomally integrated resistance element which carries the *mecA *gene, encoding the alternate penicillin-binding protein PBP2a, which confers methicillin resistance. There are currently six main types of SCC*mec*, differentiated according to their combinations of *mec *complex, containing the *mecA *gene and various portions of its regulatory genes *mecR1 *and *mecI*, and *ccr *complex containing recombinases specific for the chromosomal integration and excision of the SCC*mec*. Further sub-typing is based on the presence of certain additional genes or resistance determinants within the J (so called junkyard) regions J1, J2 and J3 of the element [12]. A number of non *mecA*-encoding SCC elements, sharing some common features with various SCC*mec*s, have also been discovered in methicillin sensitive *Staphylococcus aureus *(MSSA) or coagulase-negative staphylococcal strains [13-18].

Identification of the Zurich drug clone was based on a characteristic pulsed field gel SmaI restriction pattern and the presence of a unique, previously uncharacterised SCC*mec *element which was termed SCC*mec*_N1_. In addition to methicillin resistance, all drug clone isolates were shown to be resistant to trimethoprim and most were resistant to sulfamethoxazole or to ciprofloxacin. MLST typing revealed that the representative isolate of this clone, MRSA CHE482, belonged to sequence type ST45, a genotype that has been associated with epidemic MSSA and low level oxacillin resistant MRSA, which seem to have high colonization and circulation capacities [19].

All the drug clone isolates have low oxacillin resistance levels, with MICs between 0.5 and 4 μg ml^-1^, which can make them difficult to detect by phenotypic tests. Except for the detection of the *mecA *gene, genotypic tests, which rely on identifying known features of SCC*mec*s [20,21] or SCC*mec*-chromosomal junctions [22] (X. Schneider, unpublished), have also failed to identify this clone [9].

To facilitate accurate molecular identification of this clone this manuscript presents a detailed description of the novel SCC*mec*_N1 _and describes the SCC*mec *variability observed so far between different isolates.

## Results and Discussion

### Mapping of SCC*mec*_N1_

**Figure 1:**
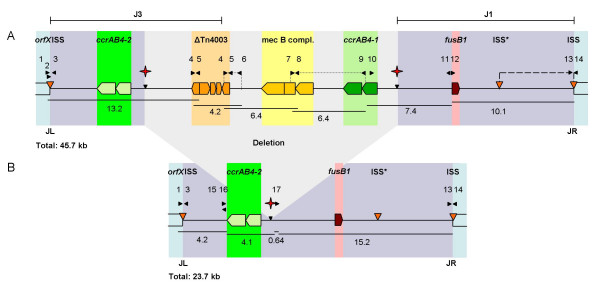
Schematic organisation of the SCC*mec*_N1 _of strain CHE482. Primers are indicated by black arrows. Regions coloured in blue represent the core chromosome. Red stars indicate the excision sites of the partially cured strain CHE482Δ. Orange arrows indicate the ISS sites and the internal ISS*. The *ccrAB4-1 *and *ccrAB4-2 *complexes are highlighted in light and dark green, respectively. The truncated Tn*4003 *in orange comprises IS*431*-*orf140*-*dfrA*-*thyE*-IS*431*. The class B *mec *complex in yellow includes *mecA-*Δ*mecR1*-IS*1272*. The fusidic acid resistance gene *fusB1 *is shown in pink. Dotted lines indicate regions of variability. The region absent in ZH4 and ZH43 is indicated by a dashed line. JL: junction left; JR: junction right. Positions of SCC*mec *regions J1 and J3 are shown. A: Entire SCC*mec*_N1 _in CHE482. B: Partially cured variant CHE482Δ.

The size of SCC*mec*_N1 _in CHE482 was estimated to be 45.7-kb, based on a series of overlapping long range PCR products amplified with primers shown in Figure 1 and listed in Table 1. This is larger than the community-associated SCC*mec *type IV (21–25-kb), type V (27.6-kb) and type VI (approximately 22-kb) elements, falling within the range of the classical hospital associated SCC*mec *types I-III which range in size from 34–67 kb [23]. Loci of interest within SCC*mec*_N1 _were then further mapped and sequenced.

**Table 1:** Oligonucleotide primers used in this study

Primer name	Nucleotide sequence (5'-3')	Reference
Mapping and sequencing		
1	CATACACCAAGATAGACATC	This study
2	ACAACGCAGTAACTATGCAC	This study
3	GTTTATCTTCATAGACTAAC	This study
4	TTCGATGTACAATGACAGTC	IS431R, this study
5	AAGGATGTTATCACTGTAGC	IS431F, this study
6	ATGTCCCAAGCTCCATTTTG	HVR P1 F [42]
7	ACGTGTTAAGTATATTGCAC	This study
8	AAGTAGTAGCTCAACGAGCT	This study
9	CAGACAATCACATCTAACAC	This study
10	TGTTGATTGACAGTAAGGAC	This study
11	GAGTACTATAGCGTATGATGT	fusR, this study
12	ACAAACGATATGAATTCCCA	fusF, this study
13	GTTTATCTTCATAGACTAAC	This study
14	CTAATATGTTGGCGCTGATAT	This study
15	CTACACTACTATTCTTTCAC	This study
16	ATAATTACGACAATGACTGT	This study
17	CGACAATAGGATCTAAAGAT	This study
Gene detection		
18	TCCAGATTACAACTTCACCAGG	MECAP4 [20]
19	CCACTTCATATCTTGTAACG	MECAP7 [20]
20	AATAGACGTAACGTCGTACT	dfrAF, this study
21	AAGAATGTATGCGGTATAGT	dfrAR, this study
Cloning		
22	ATTAGGATCCCTAGCTGATTTAATCGTTGAAG	This study
23	ATTATCTAGATAGTAAGATATAATGTTTGGG	This study
24	ATTAGGATCCGATTGATAGTATTGCAATCA	This study
25	ATTAGGATCCGTATAGGAGTGAATGAAATGG	This study
26	ATTAGGATCCATTGTGCTTGCACAATCCTT	This study
*ccrAB4-1/-2*_CHE482_		
27	CAAATGATTGAAACAGAGGT	This study
28	CACGTTTTCTACAATAACGT	This study

### *mec *complex typing

SCC*mec *typing [20] results suggested that the *mec *complex did not contain *mecI *and PCR using primers spanning IS*1272 *and Δ*mecR1 *and sequencing over the gene junction then confirmed the presence of a class B *mec *complex (*mecA*-Δ*mecR1*-IS*1272*).

### *ccr *typing

**Figure 2:**
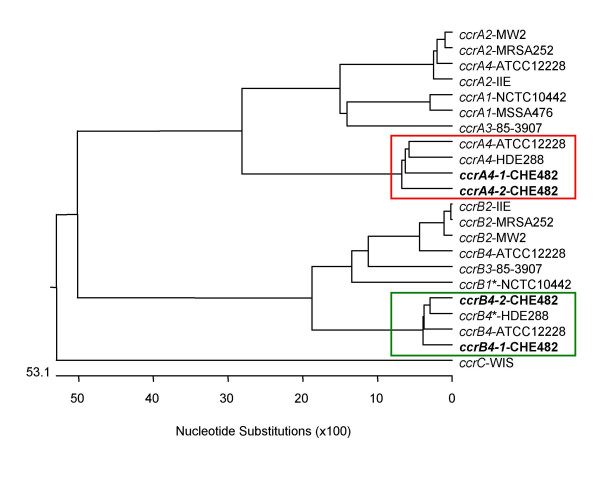
Phylogenetic relatedness of selected *ccrA *and *ccrB *nucleotide sequences. The following genes were used: *ccrA1 *and ccrB1* from strain NCTC10442 [DDBJ:AB033763]; *ccrA2 *and *ccrB2 *from MRSA252 [EMBL:BX571856], MW2 [NCBI:NC_003923], SCC*mec *IIE [EMBL:AJ810120]; *ccrA3 *and *ccrB3 *of 85–3907 [DDBJ:AB047088]; *ccrA4 *and ccrB4* from HDE288 [GenBank:AF411935]; *ccrA4 *and *ccrB4 *of ATCC12228 [GeneBank:AE015929]; *ccrC *in WIS [DDBJ:AB121219]. The *ccrB *genes from NCTC10442 (ccrB1*) and HDE288 (ccrB4*) are truncated; for comparison we have reconstituted them by adding an adenine at the site of the frameshifts. The evolutionary relationships are shown by the length of the branches and the scale of the tree indicates the number of nucleotide substitutions per 100 bases. Alignment was done using ClustalW and tree constructed with Multalign, Lasergene 6.0. Type 4 *ccrA *genes are framed in red and and type 4 *ccrB *genes in green.

No *ccr *complex could be detected using the multiplex PCR for *ccr *types 1 to 3 as described by Ito et al. [21], however with *ccr *type 4-specific primers C1 and C2, a weak amplificate was produced [9]. Further sequence analysis revealed that this SCC*mec *contained two complete *ccrAB *loci which are both similar to *ccrAB4 *from strain HDE288, a pediatric clone isolated in Portugal carrying a type VI SCC*mec *element [24,25]. Therefore specific primers to identify the drug clone *ccrAB4-1/-2 *genes were designed (primers 27 and 28). One of the loci, *ccrAB4-1*, was located at the usual *ccrAB *position downstream of the *mec *complex at the border of the J1 region. The other recombinase complex, *ccrAB4-2 *was located within the J3 region (Figure 1). Sequence alignments of *ccrAB4-1*_CHE482_, *ccrAB4*-2_CHE482_, *ccrAB4*_HDE288 _and *ccrAB4*_ATCC12228 _genes showed that all four loci were different, with *ccrA4 *genes sharing between 85.2% and 89.4% similarity with each other and *ccrB4 *sequences sharing between 94.3% and 92.9% similarity. Nucleotide sequence similarities of these four *ccrA4 *genes and *ccrA *genes from complex types 1–3, and of the four *ccrB4 *genes with *ccrB *genes from complexes 1–3, are shown by phylogenetic tree (Figure 2). For these alignments the sequence of *ccrB4*_HDE288 _was adjusted because the database sequence is truncated as the result of an adenine deletion at nt position 1325; leaving it 99-aa shorter than *ccrB4-1*_CHE482 _and 100-aa shorter than *ccrB4-2*_CHE482_. By adding back this adenine we could compare the whole length sequences.

The phylogenetic trees show that *ccrAB4-1*_CHE482_, *ccrAB4-2*_CHE482_, *ccrAB4*_HDE288 _and *ccrAB4*_ATCC12228 _(*S. epidermidis*) form a distinct *ccrAB4 *cluster. The presence of two complete *ccrAB4 *loci in the CHE482 SCC*mec *indicated that SCC*mec*_N1 _had been composed from at least two different complete or partial SCC elements. Other such mosaic or composite SCC elements have been described previously [13,26,27], however this is the first SCC*mec *found to contain two copies of the same *ccr *complex.

Due to the presence of both a class B *mec *complex and *ccrAB4*, the CHE482 SCC*mec *would be most closely related to SCC*mec *type VI. However, due to a number of unique features, including the presence of a second *ccr *locus and additional antibiotic resistance determinants, it appears to be a distinct subtype of this group that we are provisionally calling SCC*mec*_N1_.

### Additional resistance determinants

In addition to *mecA*, the resistance genes *dfrA *and *fusB1*, encoding trimethoprim and fusidic acid resistance, respectively, were also found on SCC*mec*_N1_. The *dfrA *gene shared 100% nucleotide identity with *dfrA *from pSK1 (X13290.1), which confers high level trimethoprim resistance [28]. *dfrA *is carried on Tn*4003*, a generally plasmid-encoded composite transposon with the genetic organisation IS*431*-*rep*-IS*431*-*orf140-dfrA-thyE*-IS*431 *[28]. We speculate that Tn*4003 *had jumped into the SCC*mec*-associated IS*431*, hypothesised to be a hotspot for the integration of resistance determinants [29,30] (Figure 1). However, it had lost the *rep *gene (replication protein) and one of its flanking IS*431 *elements, leaving Tn*4003 *truncated (IS*431*-*orf140*-*dfrA*-*thyE*-IS*431*).

The *fusB1 *gene, found within the J1 region of SCC*mec*_N1_, was identical to the hypothetical fusidic acid resistance gene SAS0043 from the methicillin-susceptible strain MSSA476 [14], located on the 22.8-kb SCC-like element SCC_476, _in MSSA476. The SCC_476 _*ccrAB *genes, however, are most similar to the type 1 *ccr *complex from *S. hominis*. The *fusB1 *gene in CHE482 conferred only low level fusidic acid resistance of 6 μg ml^-1^.

### SCC*mec *boundaries

**Figure 3:**
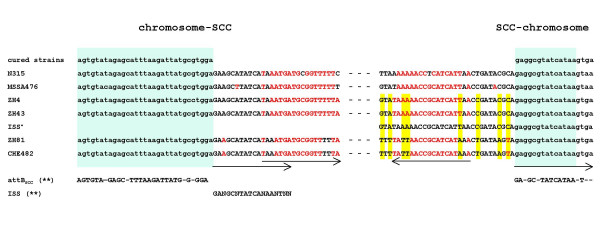
Chromosome-SCC*mec *junction sequences. The borders of strain CHE482 were aligned with the sequences of the three drug clones, ZH4, ZH43, ZH81, the cured drug clone, strain N315 (type II SCCmec), MSSA476 (SCC_476_)and ISS*. Light green boxes indicate the *orfX *region and its integration sequence site, ISS. Yellow shading highlights differences between the SCC*mec *ends. Direct and indirect repeats are indicated with arrows. The red letters represent the similarity of the inverted repeats. *attB*_SCC_** and ISS** consensus sequences were taken from Ito et al. [23].

The boundaries of the SCC*mec *element were sequenced using the primers 2, 3, 13 and 14 and compared to reference sequences of SCC_476 _from MSSA476 and SCC*mec *type II from N315 (Figure 3). SCC*mec*_N1 _had integrated at the same position within the *attB*_SCC _sequence at the 3' end of *orfX *as all previously described SCC*mec *and SCC elements. The ends of SCC*mec*_N1 _contained the characteristic direct and degenerate-inverted repeats found at the ends of SCC*mec *types I-IV and SCC_476_. Integration site sequences (ISS) with the consensus sequence 5'-(**GA**N**GC**N**TATCATAA**N**T**N)-3 [23] were present at both boundaries. A third ISS sequence (ISS*) was also identified about 6.4-kb upstream from the right end junction.

### Drug clone variability

**Figure 4:**
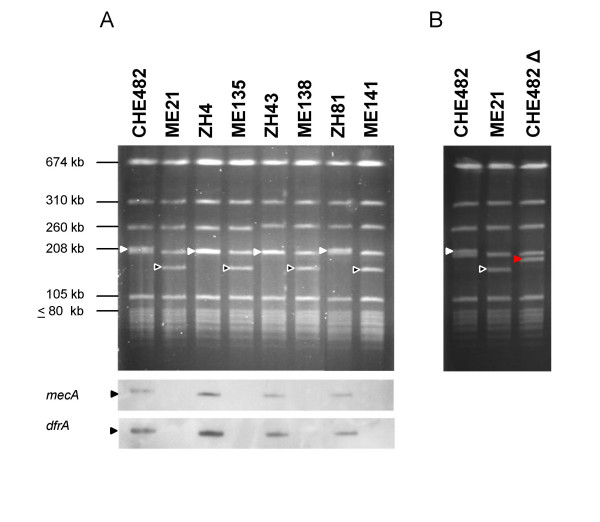
PFGE restriction analysis. A: SmaI restriction patterns of four drug clone isolates CHE482, ZH4, ZH43, ZH81 and their corresponding cured strains ME21, ME135, ME138, ME141. The SmaI fragments carrying SCC*mec *are indicated by filled triangles, and the corresponding fragments of the cured strain are indicated by open triangles. Southern hybridisations using a *mecA *and a *dfrA *probe are shown below. B: PFGE patterns of the drug clone CHE482 (filled triangle), its cured strain ME21 (open triangle) and the partially cured strain CHE482Δ (red triangle).

Analysis of PFGE profiles of all drug clone isolates characterised by Qi et al. in 2003 [9] revealed that there were small variations in size in the 208-kb SmaI band containing *mecA*. Therefore the SCC*mec *of CHE482 and a selection of three other drug clone strains (ZH4, ZH43 and ZH81, Table 2) were cured using plasmid pSR3-1. The SmaI band carrying SCC*mec *was slightly larger in CHE482 and ZH81 than in ZH4 and ZH43 before curing, whereas after curing the resulting patterns were identical in all four strains (Figure 4A). This indicated that there was variability, presumably within the SCC*mec*.

**Table 2:** Strains and plasmids

Strain	Relevant genotype	Phenotype	Origin, Reference
*S. aureus*			
CHE482	CC45, ST45, SCC*mec*_N1 _(*dfrA, fusB1*),* blaZ*	Mc^r^, Tm^r^, Fa^r^, Sx^R^	IMM collection, University Zurich
ME21	CHE482ΔSCC*mec*_N1_, *blaZ*	Mc^s^, Tm^s^, Fa^s^, Sx^R^	this study
CHE482Δ	CHE482Δ'SCC*mec*_N1 _(*fusB1*),* blaZ*	Mc^s^, Tm^s^, Fa^r^, Sx^R^	this study
ZH81	SCC*mec*_N1 _(*dfrA, fusB1*),* blaZ*	Mc^r^, Tm^r^, Fa^r^	[9]
ME141	ZH81ΔSCC*mec*_N1_, *blaZ*	Mc^s^, Tm^s^, Fa^s^	this study
ZH4	SCC*mec*_N1_(*dfrA, fusB1*),* blaZ*	Mc^r^, Tm^r^, Cp^r^, Fa^r^, Sx^R^	[9]
ME135	ZH4ΔSCC*mec*_N1_, *blaZ*	Mc^s^, Tm^s^, Cp^r^, Fa^s^, Sx^R^	this study
ZH43	SCC*mec*_N1 _(*dfrA, fusB1*), *blaZ, fusA*	Mc^r^, Tm^r^, Cp^r^, Fa^r^	[9]
ME138	ZH43ΔSCC*mec*_N1_*, blaZ, fusA*	Mc^s^, Tm^s^, Cp^r ^Fa^r^	this study
HDE288	Pediatric clone, type 4 *ccr *complex	Mc^r^	[24, 43]
*E. coli*			
DH5α	restriction-negative strain for cloning		Invitrogen
Plasmids			
pYT3	ori(ts), *S. aureus*, *tetL*	Tc^r^	[35]
pSR3-1	ori(ts) *S. aureus*, *ccrAB2 *genes, *tetL*	Tc^r^	[35]
pAW17	*S. aureus-E. coli *shuttle vector, *aac-aph*	Km^r^	[44]
pME15	pAW17 *ccrAB4-1*_CHE482_	Km^r^	This study
pME21	pYT3 *ccrAB4-1*_CHE482_	Tc^r^	This study
pME22	pYT3 *ccrAB4-2*_CHE482_	Tc^r^	This study

Abbreviations: Ap, ampicillin; CC, clonal complex; Cp, ciprofloxacin; Fa, fusidic acid; Gm, gentamicin; Km, kanamycin; Mc, methicillin; Ox, oxacillin; ST, sequence type; Sx, sulfomethoxazole; Tc, tetracycline; Tm, trimethoprim; ts, temperature sensitive.

### SCC*mec*_N1 _variation

Using the long range overlapping PCR products, variations in the SCC*mec *elements of strains CHE482, ZH4, ZH43 and ZH81, were compared. Fragment sizes between primers 6 and 4 varied by 100 to 200 bp, and between primers 7 and 9 from 1000 to 1500 bp. The variation in the hypervariable region between the *mec *complex and ΔTn*4003 *(primers 6 and 4) could be due to different numbers of direct repeats units (*dru*) [31] as found in SCC*mec *V_T _compared to the WIS SCC*mec *V [32]. Amplification between *fusB1 *and the right SCC*mec *junction produced a 6.4-kb larger end fragment from CHE482/ZH81 than from ZH4/ZH43.

Sequencing in from the right junctions showed that the ends of CHE482/ZH81 were identical to each other with no significant similarity to any database sequences (data not shown) but they were different to those of ZH4/ZH43 (Figure 3). The latter sequences of ZH4/ZH43 were identical to the end of SCC_476_. In contrast, the left end chromosome-SCC*mec *junction sequences were identical in all drug clones analysed.

### Antibiotic resistance variability

SCC*mec *variability also appeared to correlate with other strain differences. Strains ZH4 and ZH43 which had identical SCC*mec*s, were also both ciprofloxacin resistant; meanwhile CHE482 and ZH81, which share identical SCC*mec*s, were ciprofloxacin susceptible (Table 2). There was also variation in fusidic acid resistance levels. Most strains had relatively low fusidic acid resistance. ZH43, however, was highly resistant and resistance was not lost upon curing (Table 3), therefore resistance in this strain was probably additionally caused by a mutation in the chromosomal elongation factor G, EF-G (*fusA*) [33]. Since both ciprofloxacin and sulfamethoxazole resistance are chromosomal, the SCC*mec *variants found in the Zurich drug clones are very likely associated with different, closely related genetic backgrounds.

**Table 3:** Antibiotic minimal inhibitory concentration [μg ml^-1^]

strain	OX	FX	TR	FA
CHE482	1.5	8	> 32	6
ME21	0.38	4	0.38	0.125
CHE482Δ	0.38	3	0.25	6
ZH4	1.5	6	> 32	6
ME135	0.50	3	0.38	0.125
ZH43	1.5	8	> 32	> 256
ME138	0.50	3	0.50	> 256
ZH81	0.75	6	> 32	6
ME141	0.38	3	0.75	0.19
HDE288	1.5	16	0.50	NA

Abbreviations: FA, fusidic acid; FX, cefoxitin; NA, not analysed; OX, oxacillin; TR, trimethoprim.

### *ccr *activity

CHE482 was cured using either pME21 (*ccrAB4-1*) or pME22 (*ccrAB4-2*). Resulting isolates were screened for methicillin, trimethoprim and fusidic acid susceptibility and for amplification of a PCR product spanning the former SCC*mec*-chromosomal junctions (primers 1 and 14, Figure 1). Both *ccrAB4-1 *and *ccrAB4-2 *were functional and able to excise SCC*mec *even though their *ccrA *and *ccrB *amino acid sequences differed by 11.3% and 4.6%, respectively. This is consistent with the finding that several different *ccrAB *loci from type IV SCC*mec*s were all shown to be active, despite varying up to 3.7% in their amino acid sequences [34].

### Excision variants

CHE482 was cured using pSR3-1 [35], the resulting strain ME21 was sensitive to oxacillin, fusidic acid and trimethoprim (Figure 1, Table 3). During curing experiments with pME21 and pME22 we discovered that there were also many strains that had not been completely cured. One set of cured CHE482 variants maintained a fragment of 6.4-kb, and sequencing confirmed that this fragment was the portion between the ISS at the right junction and ISS*. This indicated that excision of the main SCC*mec *fragment containing all three resistance determinants had occurred through recombination between the ISS at the left junction and ISS*.

*ccrAB4-1 *and its predicted promoter were also cloned into the *E. coli-S. aureus *shuttle vector pAW17 to produce the recombinant plasmid pME15. Attempts to cure CHE482 of its SCC*mec *element using pME15 resulted in another partially cured set of variants which had maintained fusidic acid resistance but lost oxacillin and trimethoprim resistance (CHE482Δ, Figure 1B). Analysis of these strains by PFGE, showed that the SCC*mec*-containing band had become smaller but not to the same extent as the completely cured strain ME21 (Figure 4B). These results indicated that only a portion of the SCC*mec*, containing *mecA *and *dfrA*, had been lost. PCR mapping identified the location of the missing portion and sequencing over the excision sites revealed that excision was likely to be mediated by homologous recombination across regions of high nucleotide sequence similarity surrounding the two *ccr *loci, as no additional ISS sequences were found. It appeared that recombination between the two *ccr *regions resulted in the deletion of a 22-kb fragment containing *ccrAB4-1*, the class B *mec *complex and ΔTn*4003*. This recombination left an SCC-like element of 23.7-kb, which contained one *ccrAB *complex (*ccrAB4-2*) and the fusidic acid resistance determinant (Figure 1B). This truncated SCC is similar in size to the MSSA476 SCC_476 _which also contains *fusB1 *and a *ccrAB *locus, although in SCC_476 _the *ccr *genes are most similar to *ccrAB *type 1.

Therefore we have identified three possible excision variants, two resulting from the presence of three ISS, as has been seen in SCC*mec *type IV strains [26], and the third variant caused by recombination between regions of high sequence similarity.

## Conclusion

The general structure of SCC*mec*_N1 _(*ccrAB4-2, dfrA*, class B *mec *complex, *ccrAB4-1*, *fusB1*) was distinctly different from already published SCC*mec *types. Several regions of variability were found between different clinical drug clone isolates, especially in the right-end region where the presence or absence of a DNA fragment framed by ISS sequences was detected. Nevertheless this clone can now be identified by its resistance profile and its combination of class B *mec *complex and *ccrAB4 *complex sequences, thus allowing easier epidemiological identification.

## Materials and methods

### Bacterial strains and growth conditions

Bacterial strains and plasmids are listed in Table 2. The four clinical MRSA isolates CHE482, ZH4, ZH43 and ZH81 were clones associated with intravenous drug users in the Zurich area. Apart from the type strain CHE482, strains were selected from the epidemiological study in 2003 based on their PFGE patterns and resistance profiles (Table 2) [9]. Growth was at 37°C in Luria Bertani broth (Difco Laboratories, Detroit, MI, USA). Strains harbouring the temperature-sensitive plasmids pME21 or pME22 were propagated at 30°C in the presence of 10 μg ml^-1 ^tetracycline and those with plasmid pME15 were grown at 37°C in the presence of 50 μg ml^-1 ^kanamycin.

### Susceptibility testing

The minimal inhibitory concentrations (MIC) of antibiotics were determined by Etest on Mueller-Hinton agar plates (Difco Laboratories, Detroit, MI, USA) according to the manufacturer's instructions (AB Biodisk, Solna, Sweden). Disc diffusion of oxacillin, cefoxitin, fusidic acid and trimethoprim/sulfamethoxazole were done according to CLSI [36] on Mueller Hinton agar plates. Penicillinase production of cefoxitin-induced cells was assayed by nitrocefin hydrolysis and PBP2a production by the MRSA screen agglutination test from Denka Seiken (Tokyo, Japan) [37].

### SCC*mec *typing

SCC*mec *types I through IV, *ccr *types 1 to 3, and *ccr *type 4 from the pediatric clone HDE288, were identified by PCR as described by [20,21], and [38], respectively. A specific PCR was established to identify the drug clone *ccrAB4-1/-2*_CHE482 _using primer pair 27 and 28 (Table 1).

### Localization of *dfrA *and *mecA*

SmaI digested chromosomal DNA was separated by pulsed field gel electrophoresis, PFGE [39] and hybridised sequentially [40] with a *mecA *(primer pair 18 and 19) and a *dfrA *probe (primer pair 20 and 21) (Table 1).

### Cloning of the *ccrAB *genes of CHE482

Each of the two *ccrAB *complexes identified in strain CHE482, including their own promoter, were cloned into the BamHI site of the temperature-sensitive plasmid pYT3, using primers 22 and 24 for *ccrAB4-1*_CHE482 _and primers 25 and 26 for *ccrAB4-2*_CHE482_. The resulting plasmids pME21 and pME22, respectively, were electroporated into RN4220 and then transduced by Φ80α into the MRSA clinical isolates to be cured of SCC*mec*. The *ccrAB4-1*_CHE482 _was also cloned into the *E. coli-S. aureus *shuttle vector pAW17, using the primers 22 and 23 (Table 1). The resulting plasmid pME15 was then electroporated into RN4220 and transduced into the MRSA to be cured of SCC*mec*.

### Curing of SCCmec

Curing of SCC*mec *was done by the method of Katayama [35] using either the temperature-sensitive plasmid pSR3-1 containing *ccrAB *type 2 recombinase genes, or plasmids pME21 (*ccrAB4-1*) or pME22 (*ccrAB4-2*). Curing with plasmid pME15 (*ccrAB4-1*) was done by transducing the plasmid into the clinical MRSA isolates, selecting for kanamycin resistant transductants at 37°C, which were then pooled and plated on LB agar containing kanamycin, grown overnight, and screened by replica plating for loss of oxacillin resistance on 1 μg ml^-1 ^oxacillin.

### Sequence analysis

Sequencing was performed with an ABI PRISM BigDye Terminator Cycle sequencing reaction kit (US Biochemicals, Cleveland, Ohio) and an ABI Prism 310 genetic analyzer (Applied Biosystems, Foster City, California). Sequence assembly was accomplished using the DNAStar sequence analysis package (Lasergene 6.0).

Sequencing of the SCC*mec*-chromosome junctions of four drug clones was done by direct chromosomal sequencing [41] of the original and cured clones using primer 2. This nucleotide sequence provided the template for the design of primers 14, 13 and 3 (Table 1) over the chromosome-SCC*mec *junctions.

### Mapping of SCC*mec*

To estimate the size of the CHE482 SCC*mec*, long range PCR amplification of six main fragments was performed using the polymerase TaKaRa Ex Taq (TAKARA BIO INC., Shiga, Japan). PCR was done as described by the manufacturer's recommendation. Primer pairs utilised were: 1 and 5; 4 and 6; 4 and 8; 7 and 9; 10 and 11 and 12 and 14 (Table 1). Amplified PCR fragments were run against molecular weight markers (Marker II, Fermentas International, Ontario, CA; 1 kb+, Invitrogen, Carlsbad, CA) on a 0.5% agarose gel.

### Nucleotide sequence accession number

The sequences of *ccrAB4-1*_CHE482 _and *ccrAB4-2*_CHE482 _have been deposited in the GenBank (NCBI) database under accession number [GenBank: EF126185] and [GenBank:EF126186] respectively.

## Authors' contributions

ME carried out the molecular genetic and microbiological studies and drafted the manuscript. BB conceived of the study, participated in its design and helped to draft the manuscript. NM participated in the design and coordination of the study and helped to draft the manuscript. All authors read and approved the final manuscript.
